# Case Report: Twelve-year longitudinal CMR follow-up in Becker muscular dystrophy–associated cardiomyopathy

**DOI:** 10.3389/fmed.2026.1969565

**Published:** 2026-10-20

**Authors:** Mohd Imree Azmi, Masafumi Takafuji, Sven Plein

**Affiliations:** 1 Leeds Institute of Cardiovascular and Metabolic Medicine, University of Leeds, Leeds, United Kingdom; 2 Faculty of Medicine, Radiology, Universiti Kebangsaan Malaysia, Kuala Lumpur, Malaysia; 3 Department of Radiology, Mie University Hospital, Mie, Japan

**Keywords:** becker muscular dystrophy cardiomyopathy, cardiomyopathies, CMR (cardiovascular magnetic resonance), imaging, serial MRIs

## Abstract

Cardiac involvement is an important cause of morbidity and mortality in Becker muscular dystrophy. Myocardial disease may progress despite relatively limited findings on conventional echocardiography, while cardiac magnetic resonance (CMR) provides detailed assessment of ventricular function and myocardial tissue characteristics with late gadolinium enhancement (LGE), parametric mapping and strain analysis. We report a case of 57 year-old man with genetically confirmed Becker muscular dystrophy who underwent CMR examinations 12 years apart. Follow-up imaging demonstrated progressive left ventricular remodelling, a decline in left ventricular ejection fraction and interval changes in the distribution and extent of myocardial fibrosis. This case provides a long-term within-patient comparison of Becker muscular dystrophy associated cardiomyopathy and illustrates the additional information provided by serial CMR in documenting progressive myocardial involvement over time.

## Introduction

Becker muscular dystrophy is an inherited dystrophinopathy characterised by progressive skeletal muscle weakness. Cardiac involvement is an important complication and may develop independently of the severity of skeletal muscle disease. Development of dilated cardiomyopathy is an important cause of morbidity and mortality in patients with Becker muscular dystrophy (1). Although echocardiography is a useful surveillance imaging modality, cardiac magnetic resonance (CMR) is superior in terms of myocardial tissue characteristic, including the detection of myocardial fibrosis using late gadolinium enhancement (LGE). Typical LGE pattern is in the inferolateral wall of the left ventricle and follows a non-ischaemic subepicardial or midwall pattern. Advance CMR techniques may also help to characterise cardiac involvement and potentially help in the management of the disease (2). However, longitudinal CMR reports documenting changes in myocardial involvement over extended follow-up periods remain limited.

## Case presentation

A 57-year-old male patient with genetically confirmed Becker muscular dystrophy presented in 2013, at the age of 44 years, with chest pain and markedly elevated cardiac troponin levels (10,000 ng/L). Electrocardiography demonstrated right bundle branch block and invasive coronary angiography showed unobstructed coronary arteries. During subsequent follow-up, the patient reported progressive fatigue and reduced exercise tolerance.

Due to his mildly reduced left ventricular ejection fraction (LVEF) from echocardiography and recent symptoms, a cardiovascular Magnetic Resonance (CMR) was performed in 2014 on a Philips Ingenia 1.5 T (Philips Healthcare, Best, the Netherlands) to investigate the cause and assess cardiac involvement in Becker muscular dystrophy. CMR demonstrated a normal-sized left ventricle with mild concentric hypertrophy (maximum septal wall thickness 13 mm) and mild global systolic dysfunction (left ventricular ejection fraction, LVEF 49%). Late gadolinium enhancement (LGE) was qualitatively assessed by visual assessment. It showed subepicardial fibrosis involving the lateral wall (Figure 1). The right ventricle was documented as normal in size and function. Native T1 was not performed. Mild central mitral regurgitation was present. Although myocarditis was considered in the differential diagnosis, the pattern and distribution of myocardial fibrosis were typical of cardiac involvement in Becker muscular dystrophy, supporting the diagnosis of Becker muscular dystrophy associated cardiomyopathy.

**Figure 1:**
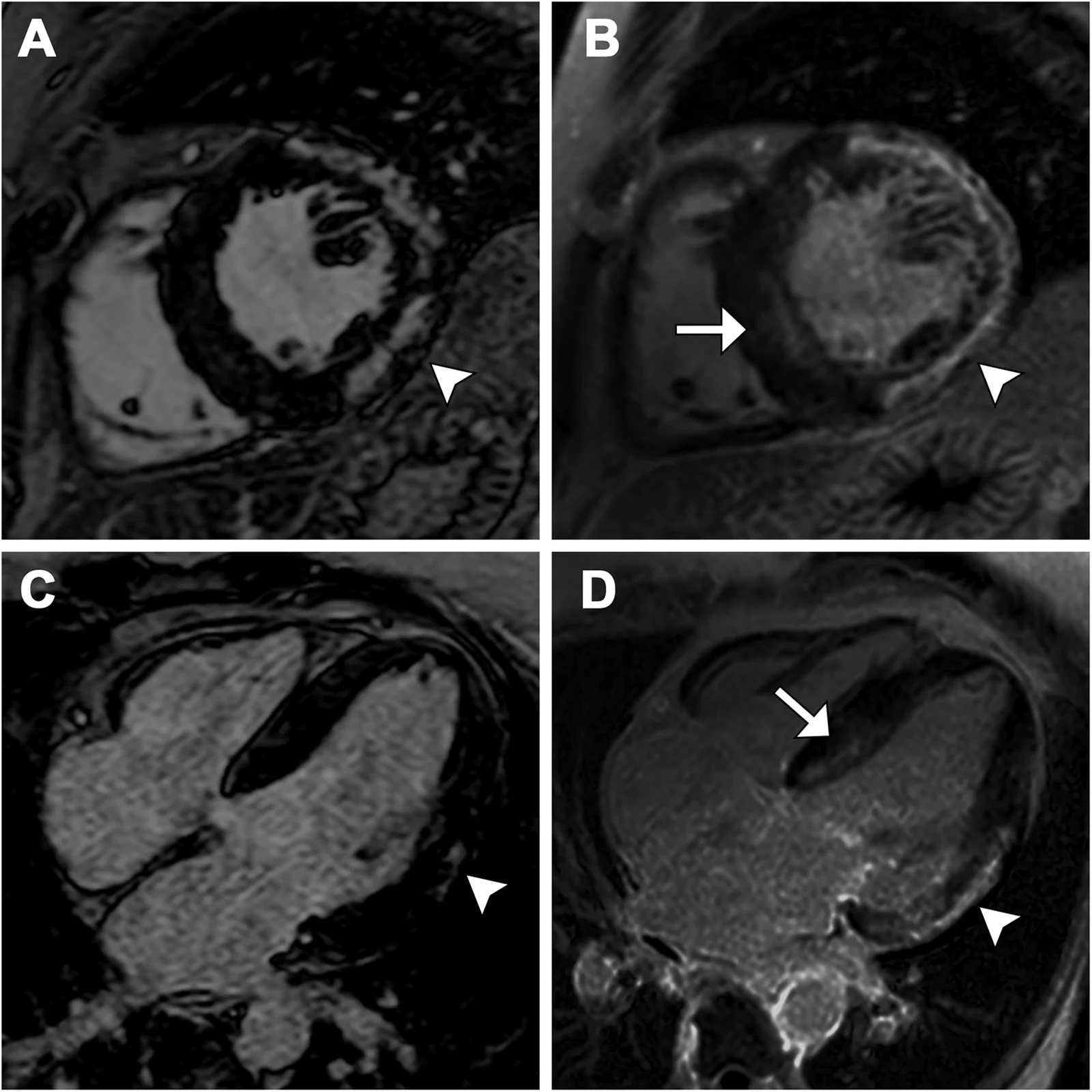
Serial late gadolinium enhancement (LGE) cardiac magnetic resonance imaging demonstrating progression of myocardial fibrosis over a 12-year interval. **(A)** Baseline short-axis LGE image. **(B)** Follow-up short-axis LGE image. **(C)** Baseline four-chamber LGE image. **(D)** Follow-up four-chamber LGE image. Arrowheads indicate progression of lateral wall subepicardial fibrosis. Arrows identify new mid-wall septal LGE on the follow-up study (B and D) showing interval progression of myocardial fibrosis.

Over the following 12 years, the patient remained under regular clinical follow-up with interval cardiac assessment (Figure 2). Serial echocardiography examinations revealed declining LVEF. In 2022, he experienced a non-ST-elevation myocardial infarction (NSTEMI) and underwent percutaneous coronary intervention with placement of a drug-eluting stent (DES), with no residual coronary artery disease. During his latest clinic visit in 2025, he had been complaining of worsening breathlessness. He denied any chest pains, palpitations or syncope. In early 2026, at the age of 57 years, he developed an episode of acute decompensated heart failure characterised by rapidly progressive dyspnoea and lower limb oedema, which improved following diuretic therapy. Transthoracic echocardiography showed a decline LVEF to 35%–40%.

**Figure 2:**
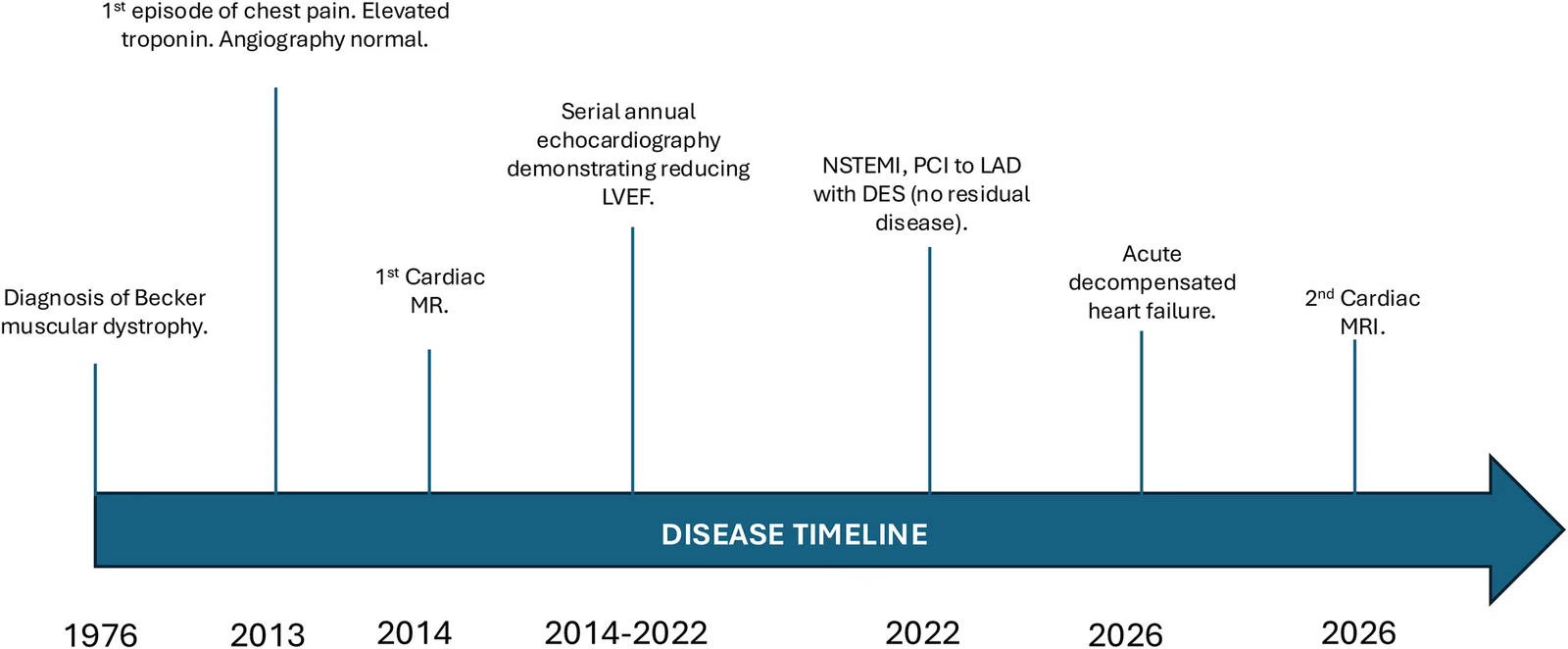
Clinical and imaging timeline. Timeline summarising the patient's clinical course and events, from the time of diagnosis in 1976, baseline CMR in 2014 and development of acute decompensated heart failure in 2026 and repeat CMR in 2026.

A repeat CMR was requested to reassess ventricular size, function and scar progression following marked declined in LVEF. The CMR in 2026, performed on a Philips Achieva 3.0 T (Philips Healthcare, Best, the Netherlands) showed significant progression of cardiac involvement (Table 1). Compared with the 2014 examination, left ventricular end-diastolic (LVED) volume increased from 199 mL (89 mL/m^2^) to 258 mL (123 mL/m^2^). Left ventricular ejection fraction reduced from 49% to 37%. Feature tracking analysis demonstrated worsening global longitudinal strain, GLS (from −14.3% to −11.5%), global circumferential strain, GCS (from −14.9% to −11.4%) and global radial strain, GRS (from 22.4% to 15.8%). Maximum septal wall thickness increased from 13 mm to 19 mm and left ventricular mass increased from 194 g (87 g/m^2^) to 226 g (108 g/m^2^).

**Table 1:** Comparison of serial cardiac magnetic resonance imaging parameters obtained in 2014 and 2026.

Parameters	2014	2026
LVEDV (mL)	199	258
LVEDVi (mL/m^2^)	89	123
LVEF (%)	49	37
LV mass (g)	194	226
LV mass index (g/m^2^)	87	108
RVEDV (mL)	Not dilated (no objective measurement documented)	219
RVEDVi (mL/m^2^)	-	104
RVEF (%)	Normal (no objective measurement documented)	38
Maximum septal wall thickness (mm)	13	19
Native T1 (ms)	-	1,360 ms (*normal range: 1,147-1,238 ms*)
LGE (visual qualitative assessment)	Subepicardial lateral wall	Progression of lateral wall fibrosis with new mid-wall septal fibrosis
GLS	−14.3%	−11.5%
GCS	−14.9%	−11.4%
GRS	22.4%	15.8%

Ventricular volumes and left ventricular mass are presented as absolute values and indexed to body surface area (BSA). Native T1 mapping was available only on the follow-up examination. LVEDV, left ventricular end-diastolic volume; LVEDVi, left ventricular end- diastolic volume index; LVEF, left ventricular ejection fraction; LGE, late gadolinium enhancement; GLS, global longitudinal strain; GCS, global circumferential strain; GRS, global radial strain.

LGE was again qualitatively analysed using visual assessment and noted the extent of myocardial fibrosis increased substantially with new mid-wall fibrosis involving the interventricular septum with diffuse contiguous subepicardial enhancement, resulting in near circumferential pattern (not shown). Despite a previous clinical history of NSTEMI, no LGE in an ischaemic distribution was identified to suggest myocardial infarction (Figure 1). Native T1 mapping was performed using modified Look-Locker inversion recovery (MOLLI) sequence. Global native myocardial T1 was elevated at 1,360 ms (institutional reference range 1,147–1,238 ms). Mild mitral regurgitation persisted.

Although the CMR examinations were performed at different magnetic strengths and scanners, the cine imaging and late gadolinium imaging acquisition were similar; the cine imaging was performed using balanced steady-state free precession sequence, acquiring in standard long-axis views and contagious short-axis stack covering the entire ventricles from basal to apex. Late gadolinium enhancement images were acquired using inversion-recovery sequence approximately 10 min following intravenous administration of gadobutrol (Gadovist; Bayer AG, Germany) at a dose of 0.15 mmol/kg. Both examinations were analysed using cvi42 CMR analysis software (Circle Cardiovascular Imaging Inc., Calgary, Canada), applying consistent ventricular contouring and feature-tracking methods. LGE distribution was qualitatively assessed visually by comparing corresponding myocardial segments. Left ventricular strain analysis was performed using a feature-tracking algorithm (3, 4). Endo- and epicardial borders of LV myocardium were manually traced in the end-diastolic phase of 2- and 4-chamber views and short-axis cine-CMR images. The software then automatically tracked the motion of in-plane tissue voxels through the entire cardiac cycle. LV global longitudinal strain (GLS) was derived from 2- and 4-chamber long-axis views cine-CMR, while LV global circumferential strain (GCS) and LV global radial strain (GRS) were derived from short axis cine-CMR.

His latest medications were ARNI (Angiotensin Receptor-Neprilysin Inhibitor) 49/51 mg BD, bisoprolol 2.5 mg OD, spironolactone 25 mg OD, and furosemide 40 mg OD. In addition, spironolactone 25 mg OD was added after his CMR examination and an echocardiography was requested to assess response to the heart failure medications. There was no recorded event after the CMR and his clinic visit.

Figure 1 shows CMR images that illustrate the progressive ventricular remodelling, worsening systolic function and increasing myocardial fibrosis over a 12-year interval. Figure 2 summarises disease timeline. Quantitative CMR parameters including strain analysis demonstrating interval changes over the 12-year follow-up are summarised in Table 1.

## Discussion

Cardiac disease remains the leading cause of mortality in Becker muscular dystrophy, making early diagnosis of myocardial involvement crucial for timely initiation of treatment. Myocardial involvement is typically subclinical before a decline in left ventricular (LV) systolic function, which is conventionally assessed by echocardiography (1). Compared to echocardiography, CMR provides superior functional assessment and allows myocardial tissue characterisation, particularly the detection of fibrosis with LGE imaging and parametric mapping. Previous studies have demonstrated LGE in up to 73% of Becker muscular dystrophy patients, most commonly with a subepicardial distribution involving the inferolateral wall (1). Furthermore, myocardial fibrosis and abnormal LV function have been identified in patients with otherwise normal electrocardiogram (ECG) and echocardiographic findings (5). Fibrotic changes may also precede overt LV dysfunction, suggesting that reliance on echocardiography alone may contribute to delayed detection of cardiac involvement (6).

Interestingly, patient's maximal ventricular septal wall thickness increased from 13 mm to 19 mm, and this should be interpreted cautiously. The consensus was that the increase in maximal septal wall thickness most likely reflected the progression of Becker muscular dystrophy. This interpretation was supported by the concurrent deterioration in the LEVF, ventricular remodeling and progression of myocardial fibrosis, suggesting an evolving myocardial disease process rather than isolated septal hypertrophy.

In our patient, the baseline CMR demonstrated mild LV systolic dysfunction (LVEF 49%) and typical subepicardial fibrosis in the lateral wall, in keeping with myocardial involvement. Over a 12-year follow up period, the subepicardial fibrosis became more extensive and new midwall fibrosis developed in the septum, accompanied by worsening LV systolic function. These findings differ from a previous report in which age and disease duration were not significantly associated with the extent of late gadolinium enhancement (LGE) in patients with Becker muscular dystrophy (7). This illustrates that progressive myocardial fibrosis and declining LV function may occur with advancing disease, highlighting the value of serial CMR surveillance. Patient's latest CMR in 2026 did not reveal any ischaemic pattern of enhancement despite having an NSTEMI episode in 2022.

Although LGE remains central for detection of myocardial fibrosis, advanced quantitative CMR biomarkers including native T1/T2 mapping, extracellular volume (ECV), and myocardial strain analysis may identify diffuse or subclinical myocardial involvement even before irreversible fibrosis becomes evident on conventional imaging. Previous studies have demonstrated elevated native T1, T2, and ECV values in Becker muscular dystrophy patients compared with healthy controls, suggesting the presence of diffuse myocardial involvement despite the absence of visible LGE (8). Importantly, ECV abnormalities have been identified in myocardial regions without visible LGE, supporting the concept that interstitial expansion may precede conventional markers of fibrosis (6). Additionally, myocardial strain analysis has demonstrated functional impairment before systolic dysfunction becomes apparent (9). This was demonstrated in our baseline study which showed a GLS of −14.3% with a slightly low LVEF (49%). Follow-up imaging 12 years apart demonstrated further reduction in GLS. These findings were accompanied by the expanding lateral wall subepicardial fibrosis, new septal midwall fibrosis and worsening in left ventricular systolic function; reflecting progressive impairment of myocardial mechanics with advancing Becker muscular dystrophy associated cardiomyopathy. It is important to note that although LVEF was only mildly reduced at baseline, the GLS was already impaired, indicating myocardial dysfunction. Over the subsequent twelve years, worsening in GLS, GCS and GRS coincided with progression of myocardial fibrosis and a decline in LVEF, although the temporal association observed in this case does not establish the causality. Earlier recognition of subclinical myocardial involvement through advanced quantitative CMR assessment may facilitate closer surveillance and timely cardioprotective intervention in Becker muscular dystrophy patients. Parametric mapping was not performed at the baseline CMR examination. On follow-up imaging, although the native T1 was prolonged, this finding is nonspecific and may reflect myocardial fibrosis, oedema or both, particularly in the context of recent cardiac decompensation. The absence of baseline native T1 measurements limits direct assessment of longitudinal changes.

Due to the rarity of the disease, longitudinal CMR studies in Becker muscular dystrophy remain limited and have generally reported shorter follow up periods (10). However, several limitations should be acknowledged. This single patient report includes only two CMR time points, precluding assessment of the timing or rate of disease progression. Differences in acquisition protocols, clinical events and treatment changes may have influenced the observed findings. Furthermore, LGE was assessed qualitatively and the absence of baseline native T1 mapping precluded longitudinal tissue characterisation comparisons. Despite these considerations, this case provides a valuable long-term evolving myocardial involvement in BMD.

## Conclusion

In conclusion, this case demonstrates a 12-year within patient CMR longitudinal comparison in Becker muscular dystrophy, demonstrating interval left ventricular remodelling, worsening systolic function and qualitative myocardial fibrosis distribution. This case highlights the value of serial CMR in documenting myocardial involvement over time in Becker muscular dystrophy, although their broader clinical implications require further investigation.

## Data Availability

The original contributions presented in the study are included in the article/Supplementary Material, further inquiries can be directed to the corresponding author.
